# IGF-1 receptor regulates dynamic changes in neuronal polarity during cerebral cortical migration

**DOI:** 10.1038/s41598-017-08140-5

**Published:** 2017-08-09

**Authors:** Alvaro F. Nieto Guil, Mariana Oksdath, Linnea A. Weiss, Diego J. Grassi, Lucas J. Sosa, Marta Nieto, Santiago Quiroga

**Affiliations:** 1 Departamento de Química Biológica-CIQUIBIC, Fac.de Ciencias Químicas, Universidad Nacional de Córdoba, CONICET, Córdoba, X5000HUA Córdoba Argentina; 2 Department of Molecular and Cellular Biology, Centro Nacional de Biotecnología, CSIC (CNB-CSIC), Darwin 3, Campus de Cantoblanco, Madrid, 28049 Spain; 3 Present Address: Department of Immunology and Microbial Science, The Scripps Research Institute, Jupiter, Florida USA

## Abstract

During cortical development, neurons undergo polarization, oriented migration and layer-type differentiation. The biological and biochemical mechanisms underlying these processes are not completely understood. In neurons in culture we showed that IGF-1 receptor activation is important for growth cone assembly and axonal formation. However, the possible roles of the insulin like growth factor-1 receptor (IGF-1R) on neuronal differentiation and polarization *in vivo* in mammals have not yet been studied. Using *in utero* electroporation, we show here that the IGF-1R is essential for neocortical development. Neurons electroporated with a shRNA targeting IGF-1 receptor failed to migrate to the upper cortical layers and accumulated at the ventricular/subventricular zones. Co-electroporation with a constitutively active form of PI3K rescued migration. The change of the morphology from multipolar to bipolar cells was also attenuated. Cells lacking the IGF-1 receptor remain arrested as multipolar forming a highly disorganized tissue. The typical orientation of the migrating neurons with the Golgi complex oriented toward the cortical upper layers was also affected by electroporation with shRNA targeting IGF-1 receptor. Finally, cells electroporated with the shRNA targeting IGF-1 receptor were unable to form an axon and, therefore, neuron polarity was absent.

## Introduction

The vertebrate cortex is responsible for the high cognitive functions of the brain. It is organized into distinct layers of neurons and within each layer neurons share similar functions, morphology and birthdates^1^. This organization optimizes the processing of information and it requires the tightly regulated migration of neurons during development^2^. Most cortical pyramidal projecting neurons originate from asymmetric division of radial glia progenitors in the ventricular zone. They then migrate radially towards the marginal zone and through the subventricular zone (SVZ) and lower intermediate zone. This migration requires an intriguing intermediate step in the lower intermediate zone (IZ) where neurons transiently become multipolar and where they dynamically extend and retract multiple long projections and move in apparently random directions^2–4^. Subsequent to this stage polarity is necessary to define neuronal projections as dendrites or axons and axogenesis starts as cells approach the middle of the intermediate zone. After the axon emerges, the cells reorient their centrosomes and Golgi toward the pial surface, as they move to the upper part of the intermediate zone^5^. Their morphology then changes from multipolar to bipolar and they resume radial migration^6^. Bipolar cells have a thick, radially oriented leading process (the future principal dendrite) and a thin trailing axon, and move by locomotion plus somal or nuclear translocation along the radial glia processes^2, 7, 8^. Neuronal orientation and polarity are thought to be regulated by extracellular signals, providing instructive cues to migrating neurons^9^.

Changes in polarity and morphology have been more extensively studied *in vitro*. In hippocampal neurons in culture, a particularly early event in neuronal polarization is the segregation of activatable, membrane inserted, IGF-1R to one neurite in neurons that do not yet exhibit a discernible axon (stage 2 of differentiation^10^). Subsequently, phosphatidylinositol-3 kinase (PI3K) is activated and its product, PIP3, accumulates in the distal region of the neurite, together with IGF-1R. These events are critical for the outgrowth of the future axons and the establishment of neuronal polarity^10–12^. Similarly, IGF-1R activation was reported to be necessary for the regulation of axonal outgrowth of motor neurons^13^. However, a possible role of the IGF-1R in neuronal migration and the establishment of polarity in the cortex has not been addressed.

Here, we show that the IGF-1R regulates the migration of cortical pyramidal neurons. Neurons electroporated with a shRNA targeting IGF-1R (shRNA-*IGF-1R*) fail to migrate to the upper cortical layers and accumulate at the ventricular/subventricular zones. Co-electroporation with a constitutively active form of PI3K rescued migration. Knocking down IGF-1 abrogated the morphological change from multipolar to bipolar and cells were arrested as multipolar forming heterotopic tissue. This correlates with the disruption of the typical orientation of the Golgi complex towards the marginal zone found in control migrating bipolar neurons. The cells electroporated with the shRNA-*IGF-1R* were unable to form an axon. In summary, the results indicate a necessary role of IGF-1 signaling in migration and the dynamic changes in neuronal polarities that occur at the SVZ/IZ during cortical development.

## Results

### IGF-1R pathway regulates cortical neuronal migration

**Figure 1:**
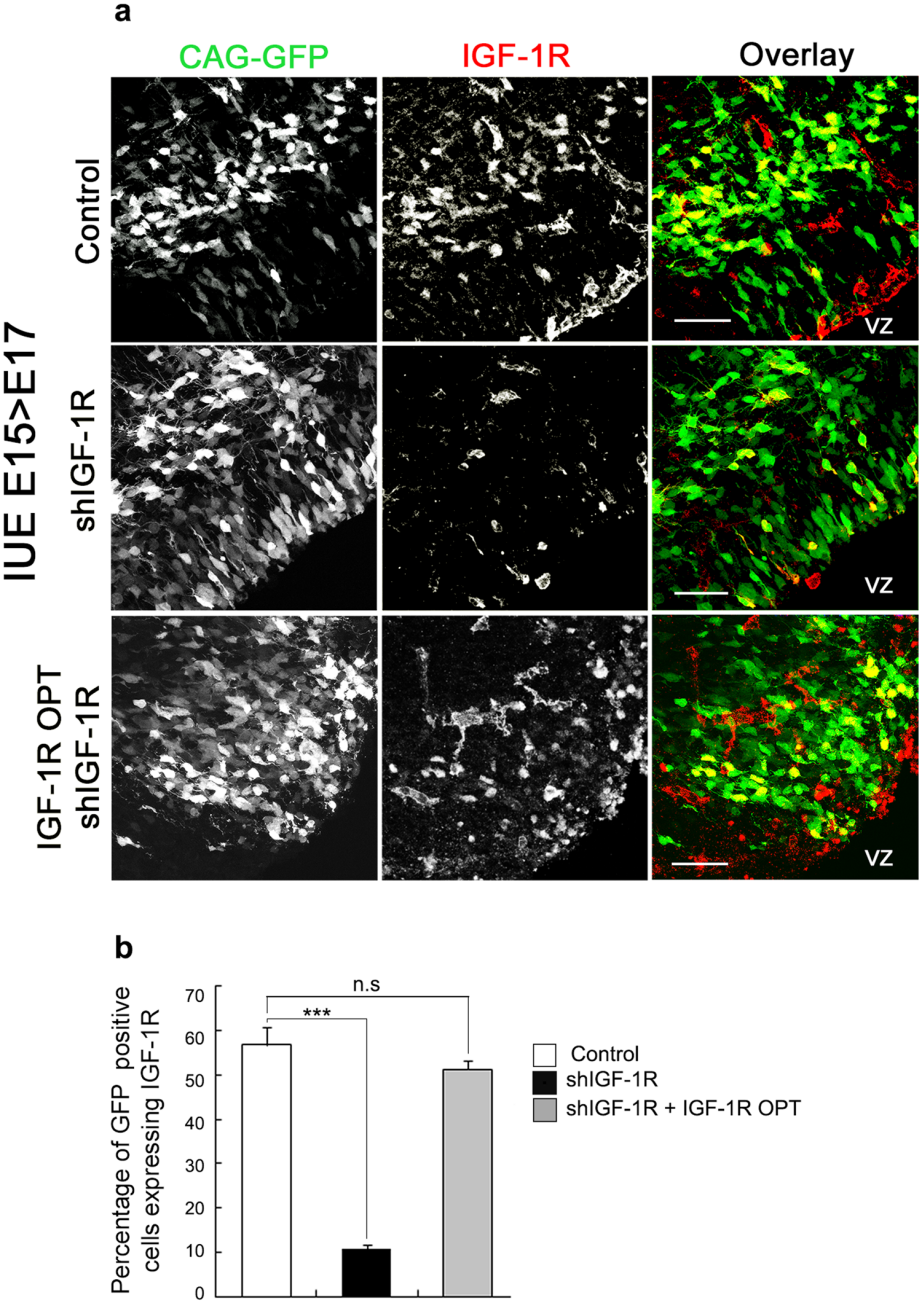
Electroporation with shRNA-*IGF-1R* (shIGF-1R) significantly reduces expression of IGF-1R. (**a**) Representative images of brains electroporated at E15 with control shRNA (top), shRNA-*IGF-1R* (middle) or co-electroporated with IGF-1R OPT and shRNA-*IGF-1R* (bottom) showing the expression of IGF-1 R (at E17). The ventricular zone (VZ) is labeled for orientation. All brains were co-electroporated with CAG-GFP. (**b**) Quantification of the number of electroporated cells positive forIGF-1R as shown in A. Note the significant decrease of IGF-1R expression in the cells electroporated with shRNA-*IGF-1R*. Student’s t test. ***p ≤ 0.0001, ns = not significant. n = 3 independent experiments. At least 100 cells were scored for each condition.

**Figure 2:**
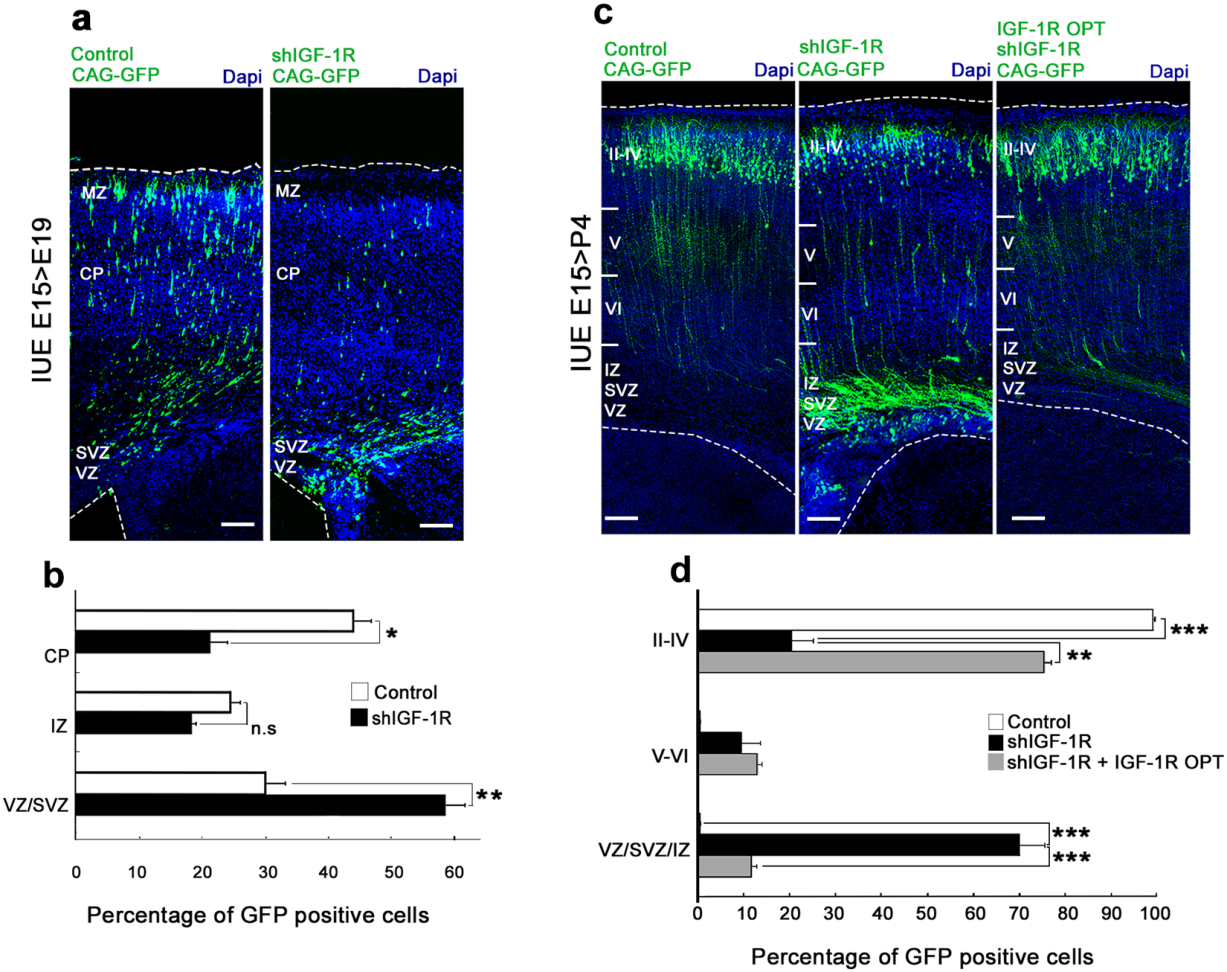
Expression of IGF-1R regulates neuronal migration. (**a**) Brains were electroporated with shRNA-*IGF-1R* (shIGF-1R/CAG-GFP) (right) or control shRNA/CAG-GFP (control-left), at E15 and analyzed at E19. Few GFP positive cells were located in the cortical plate (CP) and the marginal zone (MZ) when IGF-1R expression was knocked down compared to control. Calibration bar = 50 μm. (**b**) Quantification of the distribution of GFP-positive cells in CP, intermedial zone (IZ) and ventricular/subventricular zones (VZ/SVZ)as indicated in A. Student’s t test *p ≤ 0.05; **p ≤ 0.01; ns = not significant. (**c**) Brains were electroporated at E15 with shIGF-1R/CAG-GFP (middle), control shRNA/CAG-GFP (left) or co-electroporated with shIGF-1R/CAG-GFP plus cDNA coding for IGF-1R OPT and analyzed at P4. A few GFP positive cells are located in layers V-VI and noticeably fewer GFP positive cells are found in layers II-IV in the IGF-1R suppressed brain compared to the control. In contrast, an important accumulation of GFP positive cells was observed at the (VZ/SVZ/IZ) zones in the shRNA-IGF-1R brain. Note that co-transfection with cDNA coding for IGF-1R OPT (shRNA-refractive cDNA that will express the IGF-1R even in the presence of shRNA-*IGF-1R*) rescued the morphology. Calibration bar = 100 μm. (**d**) Quantification of the distribution of GFP positive neurons in VZ/SVZ/IZ, layers II-IV, V and VI as in c. Post hoc Turkey’s ANOVA **p ≤ 0.01 ***p ≤ 0.0001. n = 3 independent experiments. An average of 300 cells (**a**,**b**) or 500 cells (**c**,**d**) were scored for each condition.

We set out to investigate a possible role of IGF-1R in cortical migration by utilizing *in utero* electroporation of cortical progenitors at embryonic day (E) 15 in order to manipulate and visualize neurons destined to comprise layers II-IV of the cortex, allowing analysis of the location and morphology of the progeny after *in vivo* differentiation. Electroporation of shRNA-*IGF-1R* resulted in effective suppression of IGF-1R expression in most electroporated cells, as shown in Fig. 1. In brains co-electroporated with a non-relevant shRNA (control) and a plasmid encoding green fluorescent protein (CAG-GFP) and then immunostained with an antibody to the IGF-1R, 57% of the electroporated cells exhibited a strong staining (Fig. 1a-top and 1b). In contrast only 10% of the cells electroporated with the shRNA-*IGF-1R* were stained (Fig. 1a middle and 1b). Co-electroporation with resistant IGF-1R construct^14^ (IGF-1R OPT) rescued IGF-1R expression to control levels (Fig. 1-bottom). Also, the number of proliferating progenitor cells as analyzed by the incorporation of bromodeoxyuridine (BrdU) was found to be unchanged by electroporation of shRNA-*IGF-1R* compared to control (Supplementary Fig. 1a): 46.7% of the cells electroporated with control shRNA vs. 49,3% of the cells electroporated with shRNA-IGF-1 incorporated BrdU. Staining with doublecourtin to identify migrating neurons^15, 16^ showed 64.4 or 63.8% of positive cells in controls or cells electroporated with shRNA-*IGF-1R*, respectively (Supplementary Fig. 1b). We next analyzed the normal differentiation of cells at E19 or postnatal (P) day 4. At E19, about one third of the neurons were located in the ventricular zone/sub-ventricular zone (VZ/SVZ), 25% of cells were found migrating through the IZ and the majority (over 40%) had reached the top of the cortical plate (Fig. 2a-left; quantification shown in Fig. 2b). By P4, almost 100% are found located in layers II-IV of the cortex (Fig. 2c-quantification shown in Fig. 2d), as expected. Cells with knocked-down expression of IGF-1R showed altered distribution and abnormal migration at both E19 and P4. At E19, over 60% of the cells remained arrested at the VZ/SVZ/IZ compared to 30% in the control experiments (Fig. 2a,b). At P4, over 70% of the GFP positive neurons were located at the VZ/SVZ/IZ when knocking down IGF1R, compared to about 10% in controls (Fig. 2c,d). To discard the possibility of nonspecific or off-target effects of the shRNA-*IGF-1R*, we co-electroporated brains with shRNA-*IGF-1R* plus IGF-1R OPT. The results of this experiment showed that co-electroporation with IGF-1R OPT cDNA rescued migration to near normal levels, with over 80% of the cells reaching layers II-IV compared to around 20% in the brains electroporated with shRNA-*IGF-1R* alone (Fig. 2c; quantification shown in Fig. 2d). This demonstrates the specificity of shRNA-*IGF-1R*-mediated defects in migration and implicates IGF-1R in cortical migration.

**Figure 3:**
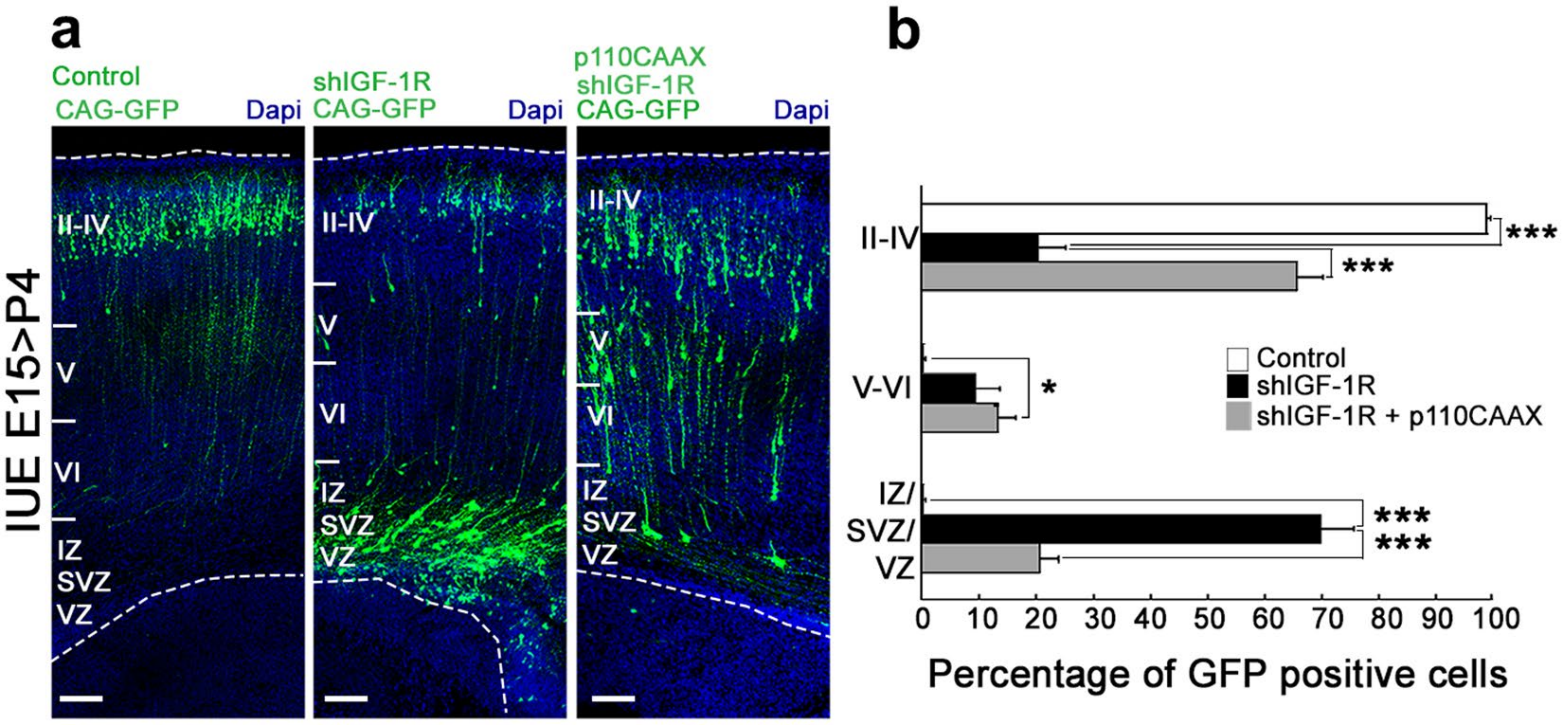
Activation of phosphatidyl inositol-3 kinase (PI3K) rescues migration defects. (**a**) Coronal sections of P4 brains electroporated at E15 with shIGF-1R/CAG-GFP, control shRNA/CAG-GFP or shIGF-1R/CAG-GFP together with a constitutively active form of PI3K (p110CAAX-right); Calibration bar = 100 μm. (**b**) Quantification of the distribution of GFP-positive cells in VZ/SVZ/IZ, and layers II-IV and V-VI as indicated in (**a**). Note the significant increase in GFP positive cells in the deep layers (VZ/SVZ/IZ) zones and the decrease of GFP positive cells in the upper layers (II-IV) when knocking down IGF-1R. Co-transfection with p110CAAX rescued migration defects Post hoc Turkey’s ANOVA **p ≤ 0.01, ***p ≤ 0,0001. n = 3 independent experiments. An average of 500 cells was scored for each condition.

IGF-1R signaling can activate the phosphatidyl inositol 3 kinase (PI3K) pathway, which promotes neurite growth and is involved in neuronal differentiation and polarization^11, 17^. To evaluate if this pathway is involved in the IGF-1R effects on the migration of cortical neurons we co-electroporated brains with shRNA-*IGF-1R* plus p110CAAX, a construct that expresses a constitutively active form of the catalytic subunit of PI3K. Analysis at P4 demonstrated rescue of migration with almost 70% of the GFP positive cells located in layers II-IV compared to 20% of cells electroporated with shRNA-*IGF-1R* alone (Fig. 3a,b). The results suggested that activation of the PI3K pathway is downstream of IGF-1R signaling and contributes to neuronal migration in the cerebral cortex.

### IGF-1R promotes axon formation in cortical neurons

**Figure 4:**
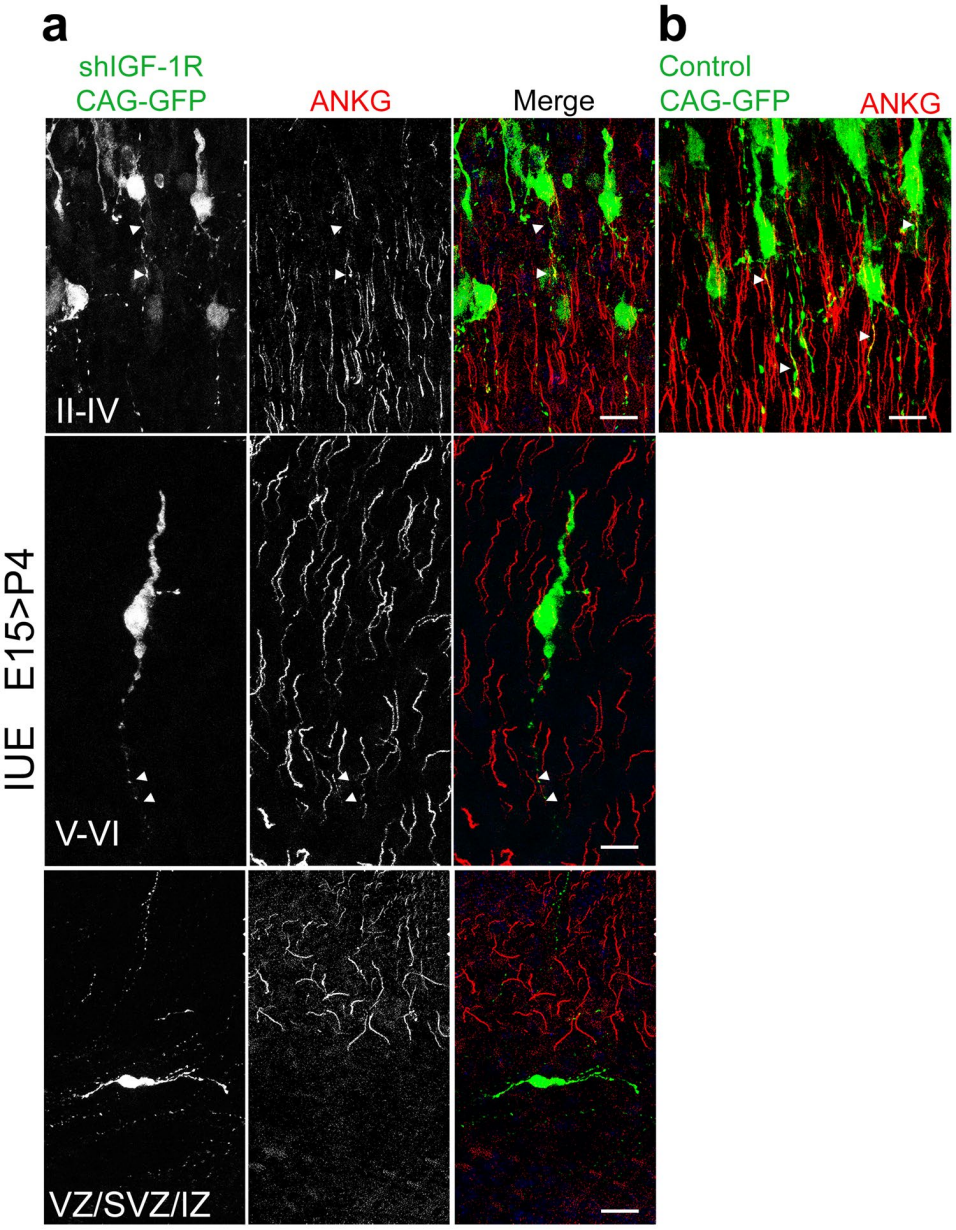
shRNA-*IGF-1R* arrested neurons fail to acquire axonal polarity as shown by ankyrin-G staining (lack of ankyrin-G clusters). (**a**) Brains were electroporated with shIGF-1R/CAG-GFP at E15 and analyzed at P4 after staining with an antibody to ankyrin-G to show axons. Cells in the V-VI layers (middle) or layers II-IV (top) show co-staining of the trailing process with ankyrin-G indicating normal polarity and the correct compartmentalization/specialization of the apical neurite into anaxonal structure. Cells arrested in the VZ/SVZ/IZ show lack of ankyrin-G staining indicating failure to develop dendritic-axonal polarity. (**b**) Brains were electroporated with control shRNA/CAG-GFP at E15 and analyzed at P4 after staining with an antibody to ankyrin-G to show axons. Cells in layers II-IV exhibit axons stained with ankyrin G. Virtually no cells were found in the VZ/SVZ/IZ and layers V-VI under this experimental condition (see Fig. 2) Calibration bar 10 μm.

We next performed ankyrin-G staining at P4 to analyze neuronal polarity and the acquisition of a mature axonal structure^18^. These immunostainings demonstrated that most cells electroporated with shRNA-*IGF-1R* arrested at the VZ/SVZ/IZ did not express ankyrin-G (Fig. 4a bottom). Only the few cells that migrate to layers II-IV (Fig. 4a top) or layers V-VI (Fig. 4 middle) exhibited an axon as shown by immunostaining for the axonal protein ankyrin-G In contrast, in control brains close to 100% of the electroporated cells migrate to layers II-IV (see Fig. 2) and develop mature axons enriched in ankyrin-G (Fig. 4b).

### IGF-1R participates in cortical neurons orientation

**Figure 5:**
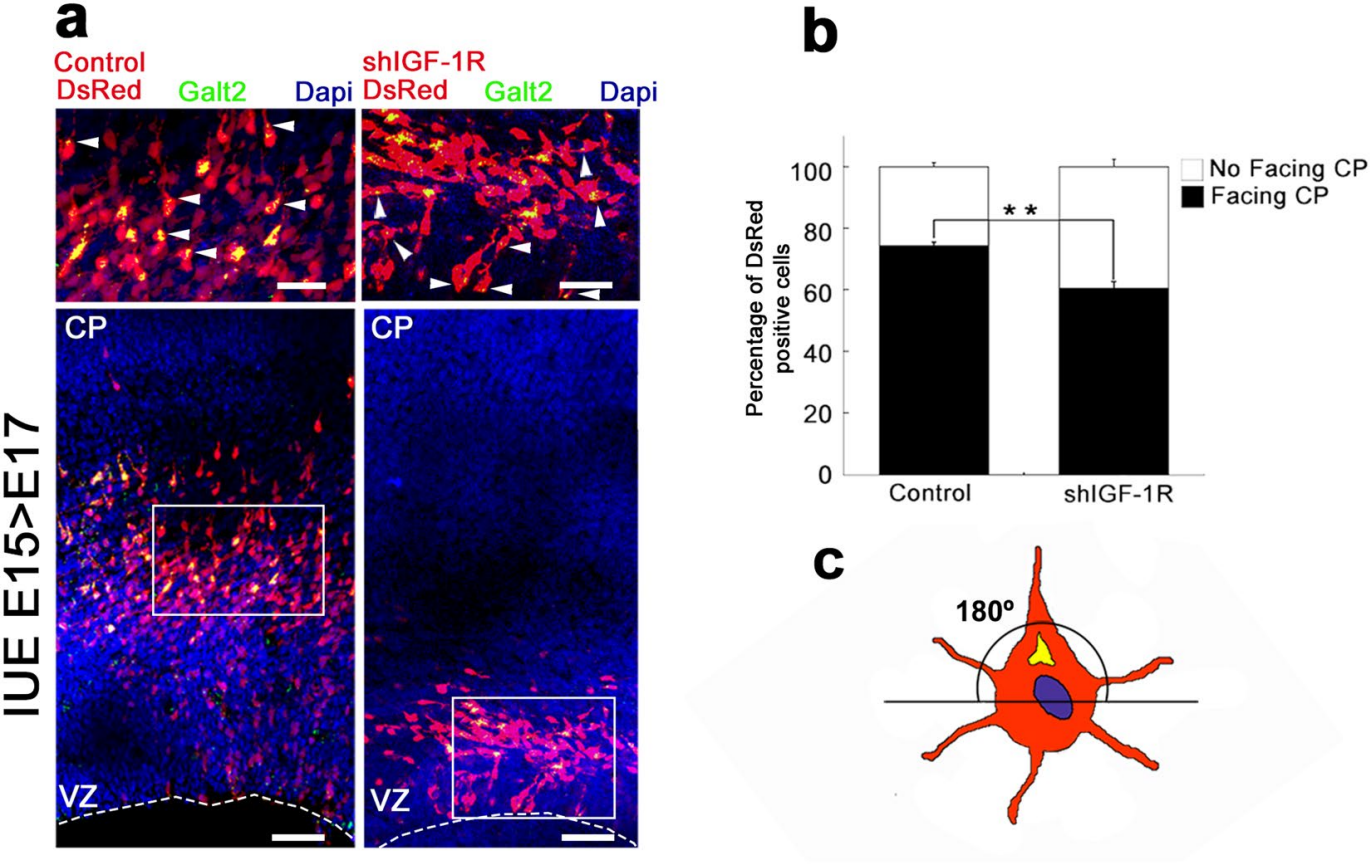
shRNA-*IGF-1R* disrupts the polarized location of migrating neurons. (**a**) Co-electroporation of shIGF-1R/DsRed and GalT2-YFP into brains at E15 (left) decreases the proportion of DsRed-positive cells in the VZ and/or IZ with a Golgi apparatus oriented towards the radial axe fate at E17 compared to a control vector (left). Lower magnification pictures (bottom) and higher magnification insets (top) are shown. Calibration bar = 50 μm (bottom) or 25 μm (top). (**b**) Quantification of the experiment shown in A. Student’s t test; **p-value = 0.01. Note that in the brains suppressed for IGF-1R the number of cells facing the cortical plate is close to 60% (random distribution = 50%). n = 3 independent experiments. An average of 300 cells was scored for each condition. (**c**) For quantifications, we considered that a cell has the Golgi not oriented to the CP when the majority of the GalT2 staining was concentrated below the axis.

We also studied early cell orientation by co-electroporating brains with a construct encoding the Golgi resident enzyme Gal-T2 tagged with yellow fluorescent protein (YFP) (Gal-T2-YFP) plus DsRed, in control and shRNA-*IGF-1R* conditions. The results of these experiments showed that, in control brains, the Golgi complex of over 75% of the DsRed positive cells were oriented towards the marginal zone (Fig. 5a left). In contrast, only 60% of the shRNA-*IGF-1R* electroporated cells exhibited the Golgi complex oriented toward the outer cortical plate, showing a close to random arrangement (Fig. 5a and b). For quantifications, we considered that a cells has the Golgi not oriented to the cortical plate when the majority of the staining was concentrated in the lower part of the axis as shown in the diagram (Fig. 5c). These experiments indicate that in the absence of IGF-1R neurons fail to maintain or acquire proper orientation.

### IGF-1R is necessary for the polarity switch in cortical neurons

**Figure 6:**
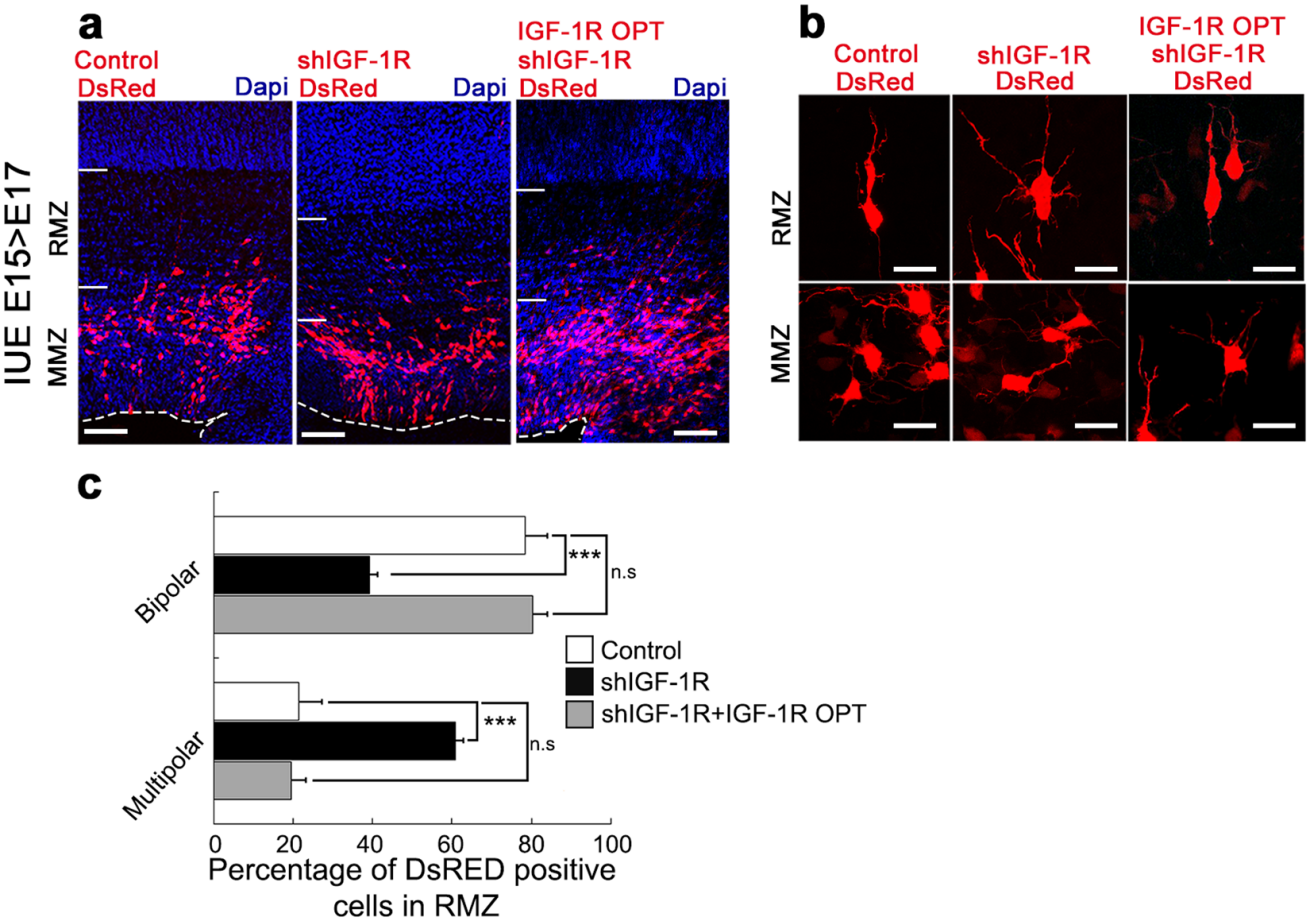
IGF-1R stimulates polarity switch of migrating neurons. (**a**) shRNA-*IGF-1R* (center) decreases the proportion of bipolar cells in the radial migration zone (RMZ) at E17 compared to a control vector (left). Co-electroporation of shRNA-*IGF-1R* and IGF-1 OPT rescued normal polarization (right). (**b**) Cell morphologies of shRNA-*IGF-1R* and control cells are shown at higher magnification at the RMZ and multipolar marginal zone MMZ Calibration bar = 50 μm. (**c**) Quantification of in the morphologies of cells located in the RMZ from a. Student’s t test; ***p-value = 0.002. n = 3 independent experiments. An average of 100 cells was scored for each condition.

Next, we studied if IGF-1R is necessary for the early transition from multipolar to bipolar morphology. Animals were electroporated at E15 and observed at E17. At this time, previous studies have shown that most cells in the lower intermediate zone and SVZ are multipolar, whereas most cells in the upper intermediate zone and cortical plate are bipolar^2–4, 19, 20^. Consequently, these regions were named the multipolar migration zone (MMZ) and the radial migrating zone (RMZ), respectively^21^. Results showed that nearly80% of control cells located in the RMZ exhibited a bipolar morphology, as defined by the absence of more than two projections (Fig. 6a,b,c). In contrast, around 60% of the shRNA-*IGF-1R* electroporated cells found in the RMZ were arrested as multipolar cells. Co-electroporation of IGF-1R OPT and shRNA-*IGF-1R* rescued normal polarization of the electroporated cells (Fig. 6a,b,c).

**Figure 7:**
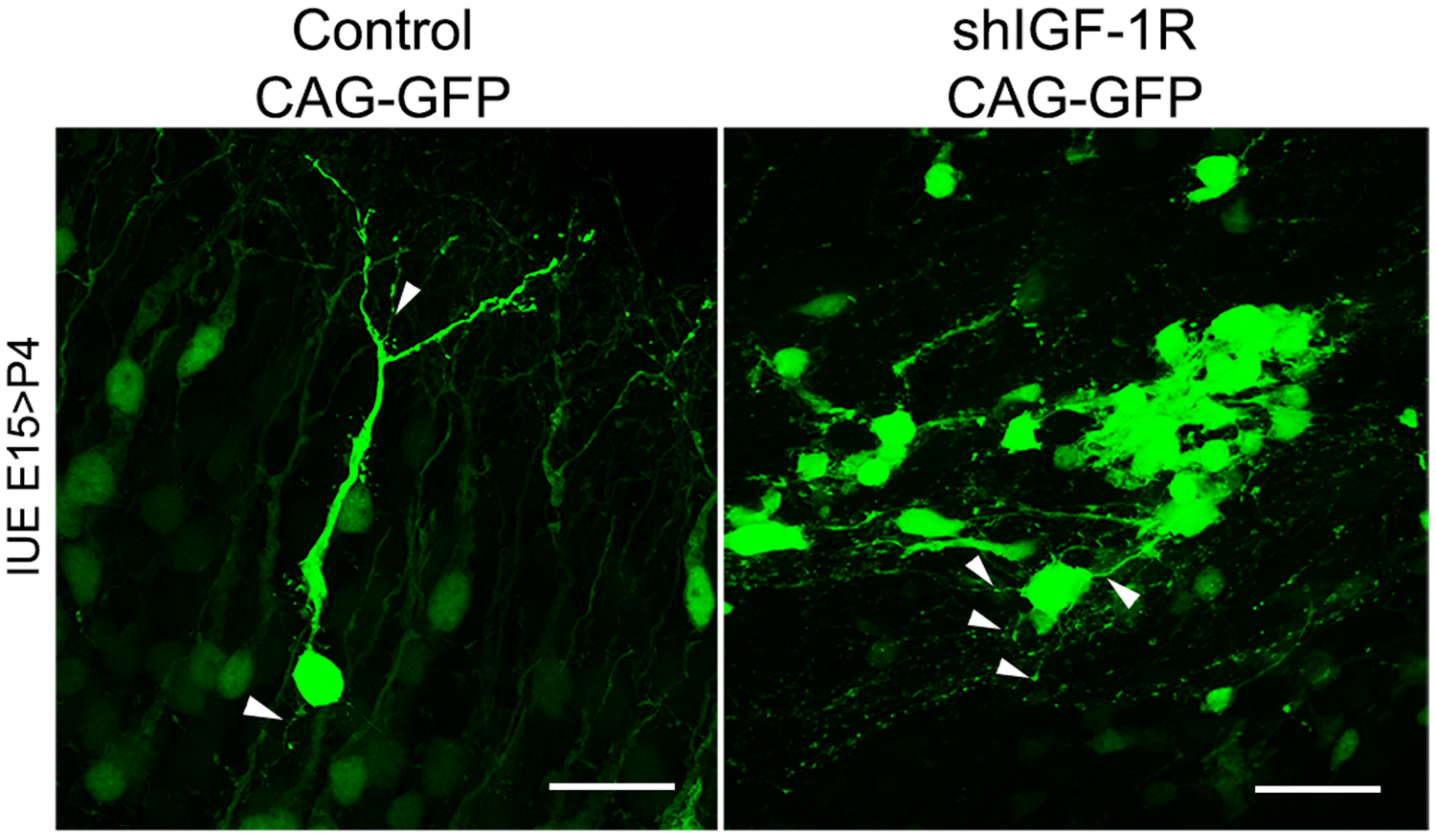
IGF-1R suppressed neurons remain as multipolar cells even at P4. Cells arrested in the VZ/SVZ/IZ in brains electroporated with the shRNA-*IGF-1R* and analyzed at P4 are mainly multipolar cells arranged in heterotopic groups (right). Control cells in layers II-IV show normal differentiation with a ramified dendrite (arrowhead) and a trailing axon (left). Calibration bar = 10 μm.

At P4, the majority of IGF-1R knocked-down cells remained arrested at the VZ/SVZ/IZ, exhibited a multipolar morphology, and clustered forming highly heterotrophic arrays (Fig. 7, right); in contrast, control cells migrated normally and exhibited a normal branched morphology of apical neurites and a tailing axon (Fig. 7, left).

## Discussion

In this work we provide evidence indicating that early expression of IGF-1R could be required for the normal orientation of cortical neuron precursors, with the Golgi complex oriented toward the cortical plate^22^. Migrating immature cortical plate neurons acquire a transient multipolar morphology in the VZ/SVZ. Then, after a polarity switch from multipolar to bipolar, they extend an axon at the upper IZ^3, 4, 19, 20^.This polarity switch is an important step during radial migration that has been implicated in specification of neuron subtype identity, cortical lamination, and projection formation^3, 9, 23–25^. Loss of function of the IGF-1R increases the proportion of neurons with multipolar morphology at the expense of bipolar cells in the RMZ. Therefore, IGF-1R is necessary for the polarity switch as well as neuronal migration, since most cells with knocked-down expression of IGF-1R remain arrested at the VZ/SVZ/IZ and are unable to form an axon, stopping neuronal polarity. We show that the IGF-1R acts through the activation of the PI3K pathway, as co- with shRNA-*IGF-1R* and a constitutively active form of PI3K rescues the migration defects.

Both *ex vivo* and *in vivo* studies have demonstrated the capacity of IGF-1 to stimulate neuronal differentiation. Studies demonstrating IGF-1 stimulation of neuritic outgrowth were among the first showing IGF-1 actions on neural cells^26^. Later IGF-1 was shown to increase dendrite growth in cultured neonatal Purkinje cells^27^ and to increase the number of pyramidal cell dendrites and their branching in somatosensory cortical explants^28^. In cultured hippocampal neurons, IGF-1 stimulates the assembly of axonal growth cones^29, 30^ and the establishment of neuronal polarity^10^. IGF-1R knock-out mice die shortly after birth and have serious defects in central nervous system development^31^. In humans, both homozygous and heterozygous mutations of the IGF-1R have been described and several developmental defects are consistently found in these patients, including microcephaly and cortical layer disorganization^32^. The PI3K pathway is also essential for neuronal polarization in hippocampal neurons in culture^11, 17^.

In summary, the results reported in this study show that IGF-1R is necessary for the early orientation and polarity switch of cortical plate neurons and, therefore, for normal neuron migration and differentiation, including axonal outgrowth and the establishment of neuronal polarity. Finally, we propose that the PI3K pathway could be involved in IGF-1R effects on cortex formation. More investigation will be needed in order to identify all the components of the IGF-1R/PI3K pathway implicated in the regulation of these phenomena.

## Methods

### Mice

Time-pregnant C57BL/6J mice were used. All animal procedures were carried out in accordance to protocols approved by the Board of Animal Welfare, School of Chemical Sciences, National University of Córdoba.

### Plasmids

shRNA plasmids: shRNA-*IGF-1R* (1) Clone identity: NM_010513.1-3300s1c1 (TRCN0000023490):5′GCAGAATAATCTAGTCCTCAT-3′ and shIGF-1R (2) Clone identity: NM_010513.1-3656s1c1 (TRCN0000023493): 5′-CCAACGAGCAAGTTCTTCGTT-3′ cloned into the plasmid pLKO 1. (Sigma Chemical Co, Mo, USA) Control shRNA does not recognize any mouse sequence. The construct pCAG-DsRed and pCAG-GFP were generous gifts from Connie Cepko. The construct IGF-1R OPT was prepared in GeneScrip (Piscataway, NJ, USA). p110CAAX was constructed at Dr. Marta Nieto Laboratory.

### In utero electroporation

In utero electroporation was performed as previously described^33^ with minor modifications. Briefly, C57BL/6J mice pregnant at E15 days were anaesthetized with isoflurane (Piramal UK). Needles for injection were pulled from P-97 Flaming/Brownglass capillaries (World Precision Instruments, Sarasota, FL, USA). shRNA solutions were mixed in 10 mM Tris, pH 8.0, Tripan blue and plasmid and injected at a concentration of 0.5–1.5 µg/µl each construct. Five pulses of 38V (50 ms ON, 950 OFF) were applied using 5 mm electrodes and a dedicated electroporator (LIADE National University of Córdoba, Argentina). The embryos were placed back into the abdominal cavity to avoid excessive temperature loss and the abdominal cavity was sutured.

### Immunohistochemistry

Mice were perfused transcardially with 4% paraformaldehyde (PFA) in PBS. The perfused brains were removed and post-fixed in 4% paraformaldehyde at 4 °C. Dissected brains were post-fixed overnight with 4% PFA in PBS. To make coronal sections, the brains were cryoprotected by overnight immersion in 30% sucrose in PBS and embedded in OCT. Floating cryosections of 50 μm were permeabilized whit PBS containing 0.5% Triton-X 100 and blocked with 2% BSA and 0.3% Triton X-100 in PBS The sections were incubated overnight at 4 °C with primary antibodies and washed with PBS, incubated with Alexa 546. (1 h at room temperature) and washed with PBS.

### Antibodies

Rat monoclonal antibody to ankyrin-G clone N106/36, NeuroMab Davis, CA, USA, diluted 1:1000; goat polyclonal antibody to doublecortin (Santa Cruz Biotechnology, Inc., CA, USA; diluted1:200; mouse monoclonal antibody to BrdU, Roche Diagnostic, Lewes, UK, diluted 1:1000; mouse monoclonal antibody to IGF-1R, clone ab80547, Abcam, Cambridge, MA, USA; diluted 1/100.

### Confocal imaging and quantification

Confocal microscopy was performed with using a confocal microscope Olympus FV1200 with Tilescan (Olympus, Japan). Images were captured and digitized using Olympus Fluoview Viewer software using a 1024 × 1024 scan format with 20x and 63x objective. All images were processed using Adobe PhotoShop (Adobe Systems, San Jose, CA, USA).

## Electronic supplementary material

Supplementary Information
