# Supplementary material for: IGF-1 receptor regulates dynamic changes in neuronal polarity during cerebral cortical migration
Source: Sci Rep. 2017 Aug 9;7:7703. doi: 10.1038/s41598-017-08140-5 (PMC5550468; doi:10.1038/s41598-017-08140-5)

IGF-1 receptor regulates dynamic changes in neuronal polarity during cerebral cortical migration.

Álvaro F. Nieto Guil^1^, Mariana Oksdath^1^, Linnea A. Weiss^2^, Diego J. Grassi^1#^, Lucas J. Sosa^1^, Marta Nieto^2^ and Santiago Quiroga^1*^

1. Departamento de Química Biológica-CIQUIBIC, Fac.de Ciencias Químicas, Universidad Nacional de Córdoba, CONICET, Argentina. 2. Department of Molecular and Cellular Biology. Centro Nacional de Biotecnología, CSIC (CNB-CSIC), Darwin 3, Campus de Cantoblanco, Madrid28049, Spain.

*Correspondence Author [squiroga@fcq.unc.edu.ar](mailto:squiroga@fcq.unc.edu.ar)

# Current address: Department of Immunology and Microbial Science, The Scripps Research Institute, Jupiter, Florida, U.S.A.

Supplementary Figure


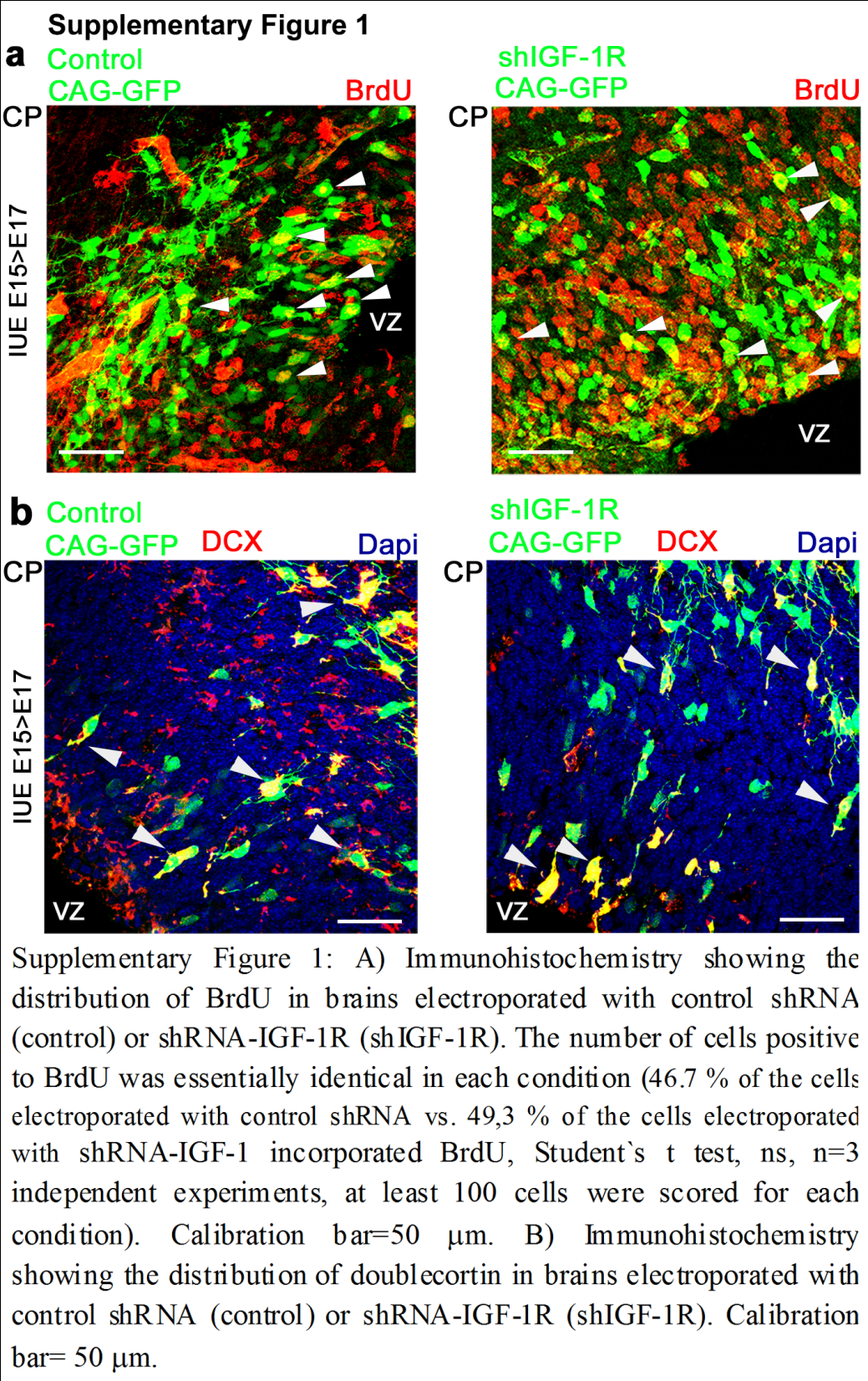

Supplement: Supplementary file 1 — Supplementary Information [file 41598_2017_8140_MOESM1_ESM.doc]
